# Effects of dulaglutide on endothelial progenitor cells and arterial elasticity in patients with type 2 diabetes mellitus

**DOI:** 10.1186/s12933-022-01634-1

**Published:** 2022-10-03

**Authors:** Dandan Xie, Yutong Li, Murong Xu, Xiaotong Zhao, Mingwei Chen

**Affiliations:** Department of Endocrinology, The First Affiliated Hospital of Anhui Medical University, No.218, Jixi Road, Shushan District, Hefei, 230032 Anhui People’s Republic of China

**Keywords:** Glucagon-like peptide-1 receptor agonists, Dulaglutide, Type 2 diabetes mellitus, Endothelial progenitor cells, Nitric oxide, Inflammatory factors

## Abstract

**Background:**

Randomised controlled trial showed that dulaglutide can reduce the risk of atherosclerotic cardiovascular disease (ASCVD) in patients with type 2 diabetes mellitus (T2DM), but the underlying mechanisms remain unclear. This study aimed to investigate the effect of dulaglutide on the number and function of endothelial progenitor cells (EPCs) in the peripheral blood of patients with T2DM and its role in improving arterial elasticity, so as to determine potential mechanisms of preventive effect of dulaglutide on ASCVD.

**Methods:**

Sixty patients with T2DM were treated with 1000 mg/day of metformin and randomly divided into two groups for 12 weeks: metformin monotherapy group (MET group, n = 30), and metformin combined with dulaglutide group (MET-DUL group, n = 30). Before and after treatment, the number of CD34^+^CD133^+^KDR^+^ EPCs and the brachial–ankle pulse wave velocity (baPWV) of the participants were measured, and EPC proliferation, adhesion, migration, and tubule formation were assessed in vitro.

**Results:**

There were no significant differences in the number and function of EPCs and baPWV changes in MET group (*P* > 0.05). In MET-DUL group, nitric oxide (NO) levels and the number of EPCs increased after treatment (*P* < 0.05), while the levels of C-reactive protein (CRP), interleukin-6 (IL-6), tumour necrosis factor-α (TNF-α), advanced glycation end products (AGEs), and baPWV decreased (*P* < 0.05). EPC proliferation, adhesion, migration, and tubule formation abilities were significantly enhanced (*P* < 0.05). Correlation analysis showed that in MET-DUL group, the changes in CRP, IL-6, TNF-α, and AGEs were negatively correlated with the number of EPCs and their proliferation and migration abilities (*P* < 0.05). Body weight, NO, CRP, and IL-6 levels were independent factors affecting the number of EPCs (*P* < 0.05). The changes in number of EPCs, proliferation and migration abilities of EPCs, and NO and IL-6 levels were independent influencing factors of baPWV changes (*P* < 0.05).

**Conclusion:**

Dulaglutide can increase the number and function of EPCs in peripheral blood and improve arterial elasticity in patients with T2DM; it is accompanied by weight loss, inflammation reduction, and high NO levels. Dulaglutide regulation of EPCs may be a mechanism of cardiovascular protection.

## Introduction

Atherosclerotic cardiovascular disease (ASCVD) is a common complication and a leading cause of death in patients with type 2 diabetes mellitus (T2DM) [1]. Studies have confirmed that ASCVD can be prevented by controlling cardiovascular risk factors in patients with T2DM [2]. Glucagon-like peptide-1 receptor agonists (GLP-1RAs) are anti-diabetic agents that improve fasting and 2 h postprandial plasma glucose; reduce glycosylated haemoglobin A1c (HbA1c), body weight, and blood pressure; and improve blood lipid profiles in T2DM patients [3], causing less hypoglycaemic events than other drugs. Dulaglutide is a long-acting GLP-1RAs formulation that is administered once weekly [4]. The Researching Cardiovascular Events with a Weekly Incretin in Diabetes (REWIND) trial showed that dulaglutide can reduce the risk of cardiovascular death, non-fatal myocardial infarction, and non-fatal stroke events in patients with T2DM and cardiovascular disease or are at a high risk of cardiovascular disease [5]. However, the mechanism of cardiovascular protective effect of dulaglutide remains unclear.

Endothelial progenitor cells (EPCs) are primordial cells derived from the bone marrow that can enter the peripheral blood circulation and differentiate into mature endothelial cells under certain conditions [6]. EPCs mainly participate in angiogenesis in ischemic tissue and repair vascular endothelial injury [7]. These processes are regulated by nitric oxide (NO), vascular endothelial growth factor (VEGF), and stromal cell-derived factor-1 α (SDF-1α) [8, 9]. Several studies have confirmed that the number and functions of circulating EPCs are highly related to cardiovascular risk factors and atherosclerosis [10]. In addition, a meta-analysis showed that lower EPC levels predicted a higher incidence of cardiovascular events, cardiovascular mortality, and all-cause mortality in patients with diabetes [11]. Studies have shown that a variety of antidiabetic drugs can not only increase the number of circulating EPCs, but also improve their function [12–14]. However, the effects of dulaglutide on the number and function of circulating EPCs in patients with T2DM remains unclear.

Arterial elasticity, also known as arterial stiffness, refers to the ratio of arterial volume to pressure. It can reflect the changes of arterial structure and function and is an independent risk factor for premature cardiovascular morbidity and mortality [15]; furthermore it can also be used to evaluate the efficacy of intervention therapy and guide medication [16]. Brachial–ankle pulse wave velocity (baPWV) is a reliable indicator of vascular elasticity and vascular injury [17, 18], and can evaluate early vascular dysfunction in T2DM patients [19]. At present, several epidemiological evidences show the predictive value of baPWV for cardiovascular events [20, 21]. It is found that GLP-1RAs can improve arterial endothelial dysfunction and stabilise atherosclerotic plaque, which is conducive to the recovery of arterial elasticity [22]. In the Sprague-Dawley rat model of non-diabetic arterial injury, glucagon-like peptide-1 receptor activation can reduce intimal hyperplasia and improve arterial wall elasticity by reducing the proliferation and increasing the apoptosis of smooth muscle cells [23]. Currently, no data on the effect of dulaglutide on vascular elasticity have been published.

In this study, we observed the effects of dulaglutide on baPWV and the number and function of EPCs in the peripheral blood of patients with T2DM. Furthermore, we analysed the possible relationship between dulaglutide and circulating EPCs and baPWV, so as to investigate the cellular biological mechanism of dulaglutide in improving vascular injury and provide a theoretical basis for determining the preventive effect of dulaglutide on ASCVD.

## Materials and methods

### Study participants

Sixty patients with T2DM, all of whom met the World Health Organisation (WHO) 1999 diagnostic criteria for diabetes, were recruited between January 2020 and April 2021 in the Department of Endocrinology at the First Affiliated Hospital of Anhui Medical University (Anhui Province, China). Inclusion criteria was as follows: age 18–65 years; male or female; diabetes duration ≤ 2 years with no ASCVD; received lifestyle intervention and 1000 mg/day of metformin (Sino-American Shanghai Squibb Pharmaceutical Co., Ltd, approval No. H20023370) for at least 8 weeks; HbA1c 7.0–7.5% (53.0–58.5 mmol/mol); body mass index (BMI) 18.5–30 kg/m^2^; and no intake of lipid-regulating drugs or a stable dose of lipid-lowering drugs for at least 6 months. Exclusion criteria were as follows: fasting C-peptide level was lower than 0.6 ng/ml; patient had acute metabolic abnormality; obvious abnormal function of heart, liver, or kidney; obvious gastrointestinal or pancreatic disease; medullary thyroid carcinoma or a family history of medullary thyroid carcinoma; serum triglyceride (TG) ≥ 5.6 mmol/l; and patient used corticosteroids, immunosuppressants, oestrogens, or exogenous cytokines, etc., in the past 6 months. This study was approved by the Medical Ethics Committee of the First Affiliated Hospital of Anhui Medical University (Ethics batch number P2020-01-12), and informed consent was obtained from all participants.

### Medication administration

Specialised personnel monitored the diet, exercise, and blood sugar levels of all participants. On the basis of maintaining the original lifestyle intervention, all participants were randomly divided into two groups: metformin monotherapy group (MET group, n = 30) and metformin combined with dulaglutide group (MET-DUL group, n = 30). The randomisation code was computer-generated by the study statistician, who assigned the next available randomisation number when informed of the next eligible participant. Participants in MET group received 2000 mg/day of oral metformin, while participants in MET-DUL group maintained the original metformin dose of 1000 mg/day and were injected subcutaneously with 1.5 mg of dulaglutide (American Eli Lilly Co., Ltd, approval No. S20190022) once weekly before breakfast. Both groups were treated continuously for 12 weeks. In the MET group, the metformin dose was reduced to the maximum tolerance dose in participants who had gastrointestinal reactions. In the MET-DUL group, if participants had obvious gastrointestinal reactions after the first injection, their dulaglutide injection could be postponed for 3 days. If the gastrointestinal reactions worsened after the second injection, participants were immediately withdrawn from the study and were offered other treatment options.

### Medical physical examination and laboratory tests

Participants fasted for 10 h in preparation for their physical examination and laboratory tests (7.00–8.00 a.m.). The height, weight, systolic blood pressure, diastolic blood pressure, and BMI defined as weight per height squared (kg/m^2^) of each participant were measured on the day of their enrolment (0 week) and 12 weeks after treatment. Laboratory tests were performed with venous blood samples drawn from the median vein of participants’ elbows. Fasting blood glucose (FPG) levels were determined by the glucose oxidase method, HbA1c was detected with the use of a high-pressure liquid chromatography (HPLC). Triglyceride (TG) and total cholesterol (TCH) levels were measured by the enzymatic method. High-density lipoprotein cholesterol (HDL-C) and low-density lipoprotein cholesterol (LDL-C) were detected by the oxidase colorimetric method; serum NO by the nitrate reductase method; C-reactive protein (CRP) by the latex-enhanced scattering immunoturbidimetry assay; tumour necrosis factor α (TNF-α) and interleukin-6 (IL-6) by the double-antibody sandwich enzyme-linked immunosorbent assay (DAS-ELISA); and serum advanced glycation end products (AGEs), VEGF, and SDF-1α by ELISA.

### Arterial elasticity measurement

Smoking, alcohol, and coffee consumption were prohibited 12 h before the test. The ambient temperature was set at 23 °C, and the participants were allowed to rest in lying position. BaPWV was measured by specialised personnel using a VP-2000 arterial elastomer (Colin Company, Komaki, Japan). The measurement time was selected on the day of enrolment (0 week) and 12 weeks after treatment, respectively.

### Determination of the number of endothelial progenitor cells in peripheral blood

Participants fasted for 10 h before 2 ml of venous blood was drawn from the median vein of their elbows and placed into the ethylenediaminetetraacetic acid anticoagulant tube for examination (7.00–8.00 a.m.) at 0 and 12 weeks. The number of EPCs positive for CD34, CD133, and KDR was determined via flow cytometry following the previously-described method [13].

### Isolation, culture, and identification of endothelial progenitor cells

Peripheral blood samples (20 ml) from participants at 0 and 12 weeks were placed in heparin tubes and diluted (1:1 in volume) with phosphate-buffered saline (PBS). Then, each sample received 10 ml of Ficoll-Paque separation solution and was centrifuged at 2000*g* and 20 °C for 10 min. The mononuclear cell layer was separated, placed in EGM-2 culture medium (Lonza, Basel, Switzerland) in fibronectin-coated plates pre-lined with fibronectin, and incubated in 5% CO_2_ at 37 °C. The morphological changes of the cells were dynamically observed under an inverted fluorescence microscope (CKX53, Olympus, Tokyo, Japan). After 2 weeks, adherent cells were incubated in 2.4 µg/ml of acetylated low-density lipoprotein solution for 1 h, which was fixed with 20 g/l paraformaldehyde, incubated in a lectin antibody solution for 1 h, and observed and photographed under the fluorescence microscope. The red and green double-stained cells were identified as EPCs (so-called ‘early EPCs’) [24].

### Endothelial progenitor cells proliferation ability (EPA) test

EPC proliferation was measured using the (3-(4,5-dimethylthiazol-2-yl)-2,5-dipheny-l-tetrazolium bromide; MTT) assay. Briefly, the adherent EPCs were digested with 0.25% trypsin and suspended in Dulbecco’s Modified Eagle Medium (DMEM) culture. The EPC solution was adjusted to 1 × 10^5^ cells/ml and cultured for 72 h in a 96-well plate. Then, the EPC solution was incubated with 10 μl MTT solution (5 mg/ml) for 4 h and with 100 µl dimethyl sulfoxide under oscillation for 10 min after discarding the supernatant. The absorbance value (OD) of each well was measured using an enzyme-labelling instrument (ELx800, Bio Tek, Vermont, USA) at 490 nm.

### Endothelial progenitor cells adhesion ability (EAA) test

EPCs’ adhesion ability (EAA) was determined using a previously-reported method [24]. Briefly, the adherent EPCs were digested with 0.25% trypsin and suspended in DMEM culture medium. The EPC solution was adjusted to 1 × 10^5^ cells/ml and incubated at 37 °C for 2 h in a 96-well plate. The unadhered cells were absorbed and washed gently with PBS. Six replicate wells were prepared and performed for each sample. In each replicate, the adherent cells were counted under the microscope with five randomly-selected visual fields.

### Endothelial progenitor cells migration ability (EMA) test

The adherent EPCs were digested with 0.25% trypsin and suspended in DMEM culture medium; the concentration of EPCs was adjusted to 1 × 10^5^ cells/ml. The EPC solution (100 μl) was added to the upper Transwell chamber (CLS3412, Corning, New York, USA), while 600 μl EGM-2 medium containing 50 μg/l VEGF was added to the lower chamber. After incubation at 37 °C for 12 h, the upper chamber was removed and the culture medium was discarded. The upper chamber was rinsed twice with PBS, fixed for 15 min with 4% paraformaldehyde, air-dried at room temperature (25 °C), and dyed for 30 min with 0.1% crystal violet. The non-migrated cells at the upper chamber were wiped gently with cotton swabs, rinsed thrice with PBS, and counted under a microscope with five randomly-selected visual fields [24].

### Endothelial progenitor cells tubule formation (ETF)

The EPC solution (1 × 10^5^ cells/ml) was transferred to a 96-well plate coated with Matrigel (BD, New Jersey, USA) and incubated at 37 °C for 24 h. Tubule formation was observed and photographed under a fluorescence microscope, and the total branch length of the vascular lumen in the visual field was calculated using ImageJ software (UVP, California, USA) [24].

### Statistical methods

SPSS 19.0 software was used for statistical analyses. The measurement data were expressed as the mean standard ± deviation ($\overline{x}$ ± SD), and the paired sample t-test was used for comparison of values before and after treatment in each group. The numerical data were expressed as percentages and tested using the Chi-square (χ^2^) test. Pearson correlation analysis and multiple linear stepwise regression analysis were used to investigate the related factors affecting the number and function of EPCs before and after treatment with dulaglutide. These analyses were also used to understand the effects of clinical factors on baPWV changes to evaluate the predictive value of EPCs’ quantitative and functional changes on arterial elasticity changes. The change value (Δ-value) before and after treatment was calculated according to the index values observed at 0 and 12 weeks. Statistically-significant differences were set at *P* < 0.05.

## Results

### Overall conditions of the participants

In the MET group, 28 participants completed 2000 mg/day metformin therapy for 12 weeks; two participants did not tolerate this dose and developed significant gastrointestinal symptoms such as nausea, vomiting, and diarrhoea. As a result, they received 1500 mg/day for 12 weeks. In the MET-DUL group, five participants presented varying degrees of gastrointestinal symptoms such as nausea, vomiting, loss of appetite, and diarrhoea. Four of them experienced relief after 2 weeks and completed the 12 week course of treatment; the other participant dropped out due to intolerable gastrointestinal symptoms. No hypoglycaemic events occurred in any of the participants during the study period.

### Comparisons in baseline data between the metformin monotherapy group and the metformin–dulaglutide group

No statistically-significant differences were found in sex ratio, age, course of diabetes, BMI, blood glucose, blood pressure, blood lipid profiles, serum FCP, NO, VEGF, SDF-1α, CRP, TNF-α, IL-6, AGEs levels, number of EPCs in peripheral blood, EPA, EAA, EMA, ETF, or baPWV between the two groups at 0 week (*P* > 0.05) (Table 1).

**Table 1:** Comparisons in baseline data between the MET group and the MET-DUL group [($\overline{x}$ ± s), n (%)]

Variables	MET	MET-DUL	*P* value
Number	30	30	–
Gender male (%)	17 (56.7%)	18 (60.0%)	0.76
Age (year)	43.5 ± 4.4	42.9 ± 5.3	0.68
Diabetes duration (month)	9.3 ± 1.4	8.8 ± 1.3	0.48
BMI (kg/m^2^)	26.8 ± 2.7	27.1 ± 2.5	0.21
SBP (mmHg)	141.3 ± 14.2	139.4 ± 12.3	0.57
DBP (mmHg)	94.1 ± 11.4	93.6 ± 12.2	0.77
FPG (mmol/l)	7.4 ± 1.1	7.5 ± 0.9	0.78
FCP (ng/ml)	1.2 ± 0.3	1.1 ± 0.2	0.80
HbA1c (%; mmol/mol)	7.2 ± 0.2; 55.2 ± 1.6	7.1 ± 0.3; 54.1 ± 1.8	0.78
TG (mmol/l)	2.2 ± 0.8	2.3 ± 0.9	0.78
TCH (mmol/l)	5.7 ± 1.3	5.6 ± 1.2	0.77
HDL-C (mmol/l)	1.4 ± 0.3	1.3 ± 0.2	0.70
LDL-C (mmol/l)	3.5 ± 0.5	3.4 ± 0.3	0.76
NO (μmol/l)	68.9 ± 14.7	71.6 ± 13.9	0.31
VEGF (pmol/ml)	211.7 ± 25.8	208.3 ± 26.7	0.42
SDF-1α (pmol/ml)	2056.1 ± 512.3	1989.6 ± 531.7	0.31
CRP (mg/dl)	5.8 ± 1.1	5.7 ± 1.3	0.72
TNF-α (pg/ml)	17.9 ± 7.8	16.4 ± 7.1	0.59
IL-6 (ng/l)	126.1 ± 32.3	135.6 ± 30.8	0.29
AGEs (ng/ml)	918.9 ± 104.5	922.4 ± 106.8	0.53
EPCs (/10^6^ cell)	27.2 ± 12.3	26.1 ± 13.4	0.69
baPWV (cm/s)	1587.4 ± 103.5	1576.7 ± 111.6	0.80
EPA (OD value)	0.53 ± 0.06	0.53 ± 0.08	0.90
EAA (cell numbers)	35.95 ± 9.73	36.82 ± 10.75	0.74
EMA (cell numbers)	18.67 ± 8.32	18.89 ± 8.81	0.89
ETF (μm)	3284.21 ± 245.46	3293.78 ± 248.73	0.80

Data are presented mean ± standard deviations or numbers (%)

*MET* metformin monotherapy group, *MET-DUL* metformin combined with dulaglutide treatment group, *BMI* body mass index, *SBP* systolic pressure, *DBP* diastolic pressure, *FPG* fasting plasma glucose, *FCP* fasting C peptide, *HbA1c* glycated hemoglobin A1c, *TG* triglyceride, *TCH* total cholesterol, *HDL-C* high density lipoprotein cholesterol, *LDL-C* low density lipoprotein cholesterol, *NO* nitric oxide, *VEGF* vascular endothelial growth factor, *SDF-1α* stromal cell derived factor-1α, *CRP* C-reactive protein, *TNF-α* tumour necrosis factor-α, *IL-6* interleukin-6, *AGEs* advanced glycation end products, *EPCs* endothelial progenitor cells, *baPWV* brachial–ankle pulse wave velocity, *EPA* endothelial progenitor cells proliferative ability, *EAA* endothelial progenitor cells adhesion ability, *EMA* endothelial progenitor cells migration ability, *ETF* endothelial progenitor cells tubule forming ability

### Comparison of clinical parameters between the metformin monotherapy group and the metformin–dulaglutide group before and after treatment

**Fig. 1:**
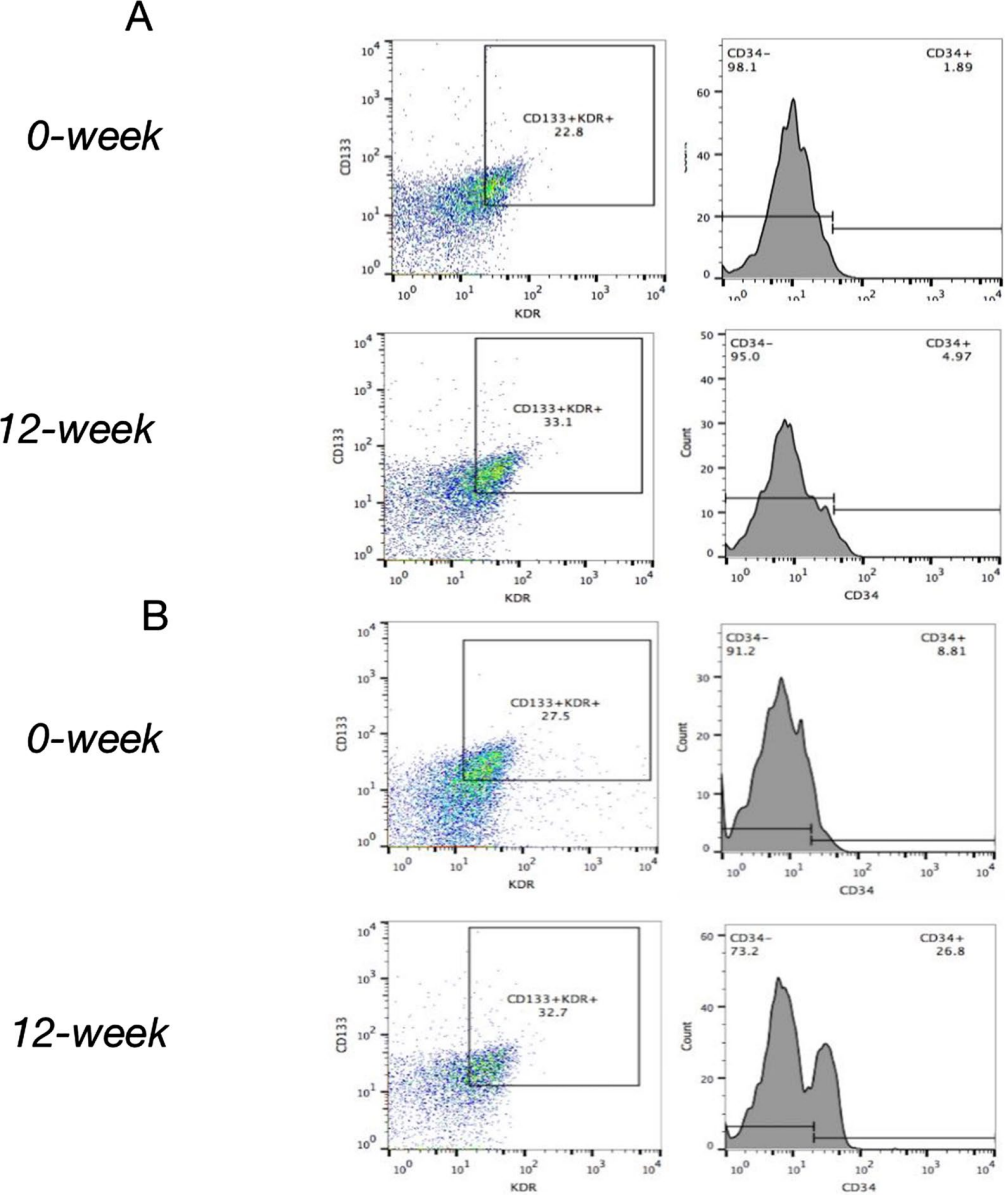
Effects of two different treatment regimens on EPCs. Representative fluorescence activating cell sorter (FACS) plots illustrating the identification of EPCs at the 0 week and after 12 weeks in a representative patient in the MET group (**A**) and the MET-DUL group (**B**). *MET* metformin monotherapy group; *MET-DUL* metformin combined with dulaglutide treatment group

**Fig. 2:**
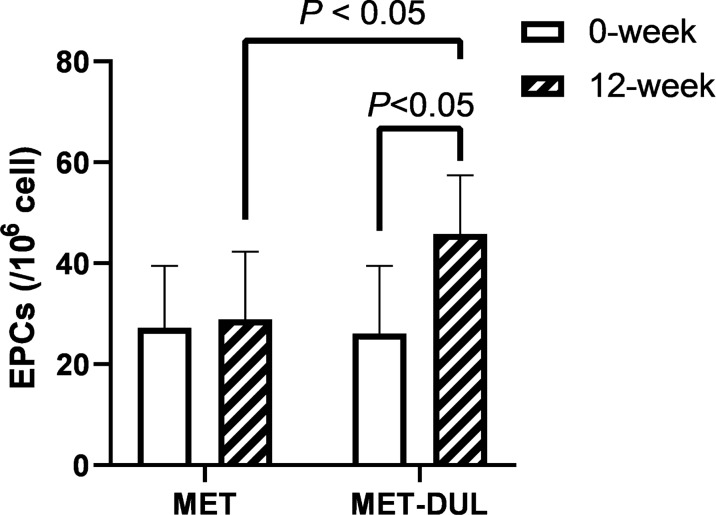
Changes in the number of EPCs before and after treatment in MET group and MET-DUL group. *MET* metformin monotherapy group; *MET-DUL* metformin combined with dulaglutide treatment group; *EPCs* endothelial progenitor cells

**Fig. 3:**
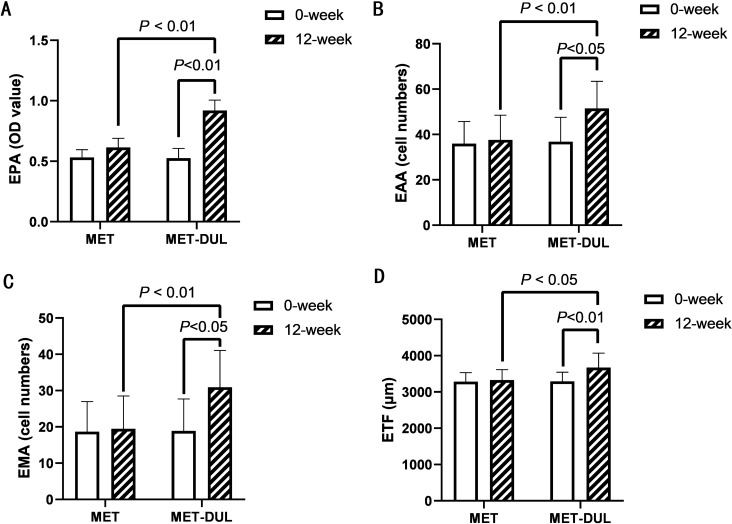
Changes in the values of EPA, EAA, EMA, ETF before and after treatment in MET group and MET-DUL group. *MET* metformin monotherapy group; *MET-DUL* metformin combined with dulaglutide treatment group; *EPA* proliferative ability of endothelial progenitor cells; *EAA* adhesion ability of endothelial progenitor cells; *EMA* migration ability of endothelial progenitor cells; *ETF* tubule forming ability of endothelial progenitor cells

**Fig. 4:**
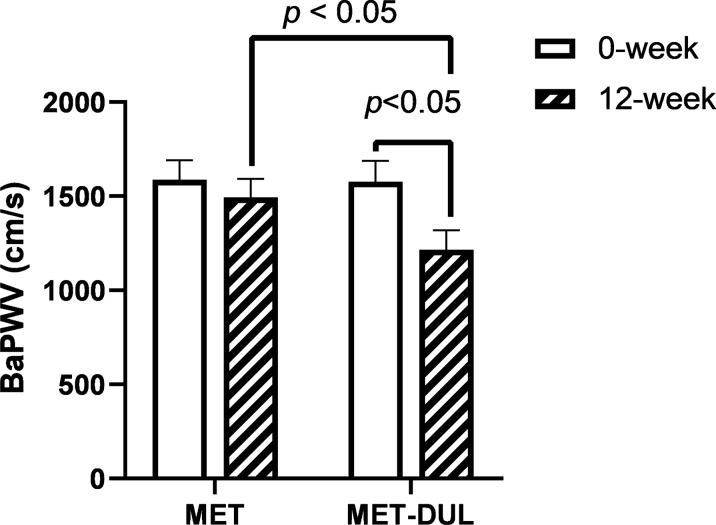
Changes in the value of baPWV before and after treatment in MET group and MET-DUL group. *MET* metformin monotherapy group; *MET-DUL* metformin combined with dulaglutide treatment group; *baPWV* brachial–ankle pulse wave velocity

In the MET group, FPG and HbA1c decreased significantly in the participants after treatment (12 weeks; *P* < 0.05), while BMI, blood pressure, blood lipid profiles, serum NO, VEGF, SDF-1α, CRP, TNF-α, IL-6, AGEs levels, number of EPCs in peripheral blood, EPA, EAA, EMA, ETF, and baPWV did not show significant differences at the end of the treatment (*P* > 0.05; Table 2, Figs. 1A, 2, 3 and 4). In contrast, in the MET-DUL group, BMI, FPG, FCP, HbA1c, CRP, TNF-α, IL-6, AGEs, and baPWV decreased significantly in the participants after treatment (*P* < 0.05), while serum NO, number of EPCs in peripheral blood, EPA, EAA, EMA, and ETF increased significantly (*P* < 0.05). However, there were no statistically significant differences in blood pressure, TCH, TG, LDL-C, HDL-C, or VEGF levels after treatment (*P* > 0.05; Table 2, Figs. 1B, 2, 3 and 4).

**Table 2:** Comparison of clinical parameters between the MET group and the MET-DUL group before and after treatment [($\overline{x}$ ± s), n (%)]

Variables	MET	MET-DUL
Number		
0-week	30	30
12-week	30	29
Male [n (%)]		
0-week	17 (56.7%)	18 (60.0%)
12-week	17 (56.7%)	17 (58.6%)
Age (year)		
0-week	43.5 ± 4.4	42.9 ± 5.3
12-week	43.5 ± 4.4	42.9 ± 5.2
BMI (kg/m^2^)		
0-week	26.8 ± 2.7	27.1 ± 2.5
12-week	26.7 ± 2.9	26.2 ± 2.6^*Δ^
SBP (mmHg)		
0-week	141.3 ± 14.2	139.4 ± 12.3
12-week	140.5 ± 15.1	136.8 ± 13.4
DBP (mmHg)		
0-week	94.1 ± 11.4	93.6 ± 12.2
12-week	94.0 ± 12.2	92.8 ± 11.3
FPG (mmol/l)		
0-week	7.4 ± 1.1	7.5 ± 0.9
12-week	7.0 ± 1.2^*^	6.9 ± 1.0^*^
FCP (ng/ml)		
0-week	1.2 ± 0.3	1.1 ± 0.2
12-week	1.3 ± 0.2	1.3 ± 0.3
HbA1c (%; mmol/mol)		
0-week	7.2 ± 0.2; 55.2 ± 1.6	7.1 ± 0.3; 54.1 ± 1.8
12-week	6.8 ± 0.2; 50.8 ± 1.5^*^	6.6 ± 0.2; 47.5 ± 1.6^*^
TG (mmol/l)		
0-week	2.2 ± 0.8	2.3 ± 0.9
12-week	2.0 ± 0.9	1.9 ± 1.0
TCH (mmol/l)		
0-week	5.7 ± 1.3	5.6 ± 1.2
12-week	5.6 ± 1.2	5.5 ± 1.1
HDL-C (mmol/l)		
0-week	1.4 ± 0.3	1.3 ± 0.2
12-week	1.4 ± 0.4	1.4 ± 0.3
LDL-C (mmol/l)		
0-week	3.5 ± 0.5	3.4 ± 0.3
12-week	3.5 ± 0.4	3.2 ± 0.4
NO (μmol/l)		
0-week	68.9 ± 14.7	71.6 ± 13.9
12-week	74.6 ± 13.9	148.9 ± 18.6^#☆^
VEGF (pmol/ml)		
0-week	211.7 ± 25.8	208.3 ± 26.7
12-week	219.5 ± 22.7	222.7 ± 27.1
SDF-1α (pmol/ml)		
0-week	2056.1 ± 512.3	1989.6 ± 531.7
12-week	2079.4 ± 496.5	2098.5 ± 548.3
CRP (mg/dl)		
0-week	5.8 ± 1.1	5.7 ± 1.3
12-week	5.7 ± 1.0	3.2 ± 1.1^*Δ^
TNF-α (pg/ml)		
0-week	17.9 ± 7.8	16.4 ± 7.1
12-week	15.6 ± 7.5	9.5 ± 5.8^*Δ^
IL-6 (ng/l)		
0-week	126.1 ± 32.3	135.6 ± 30.8
12-week	113.6 ± 29.7	88.6 ± 19.6^*Δ^
AGEs (ng/ml)		
0-week	918.9 ± 104.5	922.4 ± 106.8
12-week	883.6 ± 98.9	798.9 ± 88.4^*Δ^
EPCs (/10^6^ cell)		
0-week	27.2 ± 12.3	26.1 ± 13.4
12-week	28.9 ± 13.4	45.8 ± 11.6^*Δ^
BaPWV (cm/s)		
0-week	1587.4 ± 103.5	1576.7 ± 111.6
12-week	1493.8 ± 99.4	1215.4 ± 103.8^*Δ^
EPA (OD value)		
0-week	0.53 ± 0.06	0.53 ± 0.08
12-week	0.60 ± 0.07	0.92 ± 0.09^#☆^
EAA (cell numbers)		
0-week	35.95 ± 9.73	36.82 ± 10.75
12-week	36.23 ± 10.90	51.51 ± 11.92^*☆^
EMA (cell numbers)		
0-week	17.67 ± 8.32	18.89 ± 8.81
12-week	18.17 ± 9.06	30.93 ± 10.14^*Δ^
ETF (μm)		
0-week	3284.21 ± 245.46	3293.78 ± 248.73
12-week	3325.46 ± 287.91	3672.82 ± 395.18^#Δ^

Data are presented mean ± standard deviations or numbers (%)

*MET* metformin monotherapy group, *MET-DUL* metformin combined with dulaglutide treatment group, *BMI* body mass index, *SBP* systolic pressure, *DBP* diastolic pressure, *FPG* fasting plasma glucose, *FCP* fasting C peptide, *HbA1c* glycated hemoglobin A1c, *TG* triglyceride, *TCH* total cholesterol, *HDL-C* high density lipoprotein cholesterol, *LDL-C* low density lipoprotein cholesterol, *NO* nitric oxide, *VEGF* vascular endothelial growth factor, *SDF-1α* stromal cell derived factor-1α, *CRP* C-reactive protein, *TNF-α* tumor necrosis factor-α, *IL-6* interleukin-6, *AGEs* advanced glycation end products, *EPCs* endothelial progenitor cells, *baPWV* brachial–ankle pulse wave velocity, *EPA* endothelial progenitor cells proliferative ability, *EAA* endothelial progenitor cells adhesion ability, *EMA* endothelial progenitor cells migration ability, *ETF* endothelial progenitor cells tubule forming ability

^*^P < 0.05, ^#^*P* < 0.01 relative to 0-week in each group; ^Δ^*P* < 0.05, ^☆^*P* < 0.01 relative to 12-week in the MET group

Notably, at the end of the treatment, participants in the MET-DUL group presented significantly lower levels of BMI, CRP, TNF-α, IL-6, AGEs, and baPWV, and significantly higher serum NO, number of EPCs in peripheral blood, EPA, EAA, EMA, and ETF than those in the MET group (*P* < 0.05). However, there were no statistically significant differences in blood pressure, FPG, FCP, HbA1c, TCH, TG, LDL-C, HDL-C, serum VEGF, or SDF-1α between the two groups after 12 weeks (*P* > 0.05; Table 2, Figs. 1A, 2, 3 and 4).

### Comparison of Δ-values of clinical parameters between the metformin monotherapy group and the metformin–dulaglutide group before and after treatment

Participants in the MET-DUL group presented significantly lower ΔBMI, ΔCRP, ΔTNF-α, ΔIL-6, ΔAGEs, and ΔbaPWV than those in MET group (*P* < 0.05), while ΔNO, ΔEPCs, ΔEPA, ΔEAA, ΔEMA, and ΔETF in the participants in MET-DUL group were significantly higher than those in the MET group (*P* < 0.05). The other observed indexes did not show statistically significant differences between the two groups in their change values (*P* > 0.05; Table 3).

**Table 3:** Comparisons of Δvalues of clinical parameters between the MET group and the MET-DUL group before and after treatment ($\overline{x}$ ± s)

Variables	MET (n = 30)	MET-DUL (n = 29)	*P* value
ΔBMI (kg/m^2^)	− 0.11 ± 0.08	− 0.82 ± 0.13	0.01
ΔSBP (mmHg)	− 1.08 ± 0.11	− 2.45 ± 0.27	0.29
ΔDBP (mmHg)	0.02 ± 0.04	0.03 ± 0.02	0.76
ΔFPG (mmol/l)	− 0.41 ± 0.04	− 0.52 ± 0.09	0.65
ΔFCP (ng/ml)	0.11 ± 0.05	0.19 ± 0.04	0.47
ΔHbA1c (%; mmol/mol)	− 0.39 ± 0.08; − 2.99 ± 0.13	− 0.48 ± 0.09; − 3.68 ± 0.14	0.19
ΔTG (mmol/l)	− 0.22 ± 0.07	− 0.25 ± 0.10	0.78
ΔTCH (mmol/l)	0.09 ± 0.05	0.08 ± 0.04	0.80
ΔHDL-C (mmol/l)	0.01 ± 0.01	0.01 ± 0.01	0.90
ΔLDL-C (mmol/l)	0.01 ± 0.01	0.09 ± 0.05	0.78
ΔNO (μmol/l)	6.14 ± 1.08	38.92 ± 9.83	0.00
ΔVEGF (pmol/ml)	9.18 ± 0.85	13.71 ± 6.63	0.10
ΔSDF-1α (pmol/ml)	23.95 ± 5.37	19.26 ± 6.32	0.29
ΔCRP (mg/l)	− 0.09 ± 0.02	− 2.98 ± 0.16	0.02
ΔTNF-α (pg/ml)	− 2.29 ± 0.14	− 6.48 ± 0.18	0.04
ΔIL-6 (ng/l)	− 12.45 ± 4.89	− 56.71 ± 6.35	0.03
ΔAGEs (ng/ml)	− 35.12 ± 2.28	− 124.64 ± 14.14	0.03
ΔEPCs (/10^6^ cell)	2.12 ± 0.97	19.97 ± 5.86	0.01
ΔbaPWV (cm/s)	− 94.54 ± 13.27	− 359.8 ± 15.46	0.02
ΔEPA (OD value)	0.11 ± 0.02	0.39 ± 0.04	0.03
ΔEAA (cell numbers)	2.79 ± 0.15	14.91 ± 5.25	0.02
ΔEMA (cell numbers)	1.41 ± 0.09	12.36 ± 4.17	0.02
ΔETF (μm)	58.13 ± 16.24	388.52 ± 38.56	0.04

Data are presented mean ± standard deviations

*MET* metformin monotherapy group, *MET-DUL* metformin combined with dulaglutide treatment group, *BMI* body mass index, *SBP* systolic pressure, *DBP* diastolic pressure, *FPG* fasting plasma glucose, *FCP* fasting C peptide, *HbA1c* glycated hemoglobin A1c, *TG* triglyceride, *TCH* total cholesterol, *HDL-C* high density lipoprotein cholesterol, *LDL-C* low density lipoprotein cholesterol, *NO* nitric oxide, *VEGF* vascular endothelial growth factor, *SDF-1α* stromal cell derived factor-1α, *CRP* C-reactive protein, *TNF-α* tumour necrosis factor-α, *IL-6* interleukin-6, *AGEs* advanced glycation end products, *EPCs* endothelial progenitor cells, *baPWV* brachial–ankle pulse wave velocity, *EPA* endothelial progenitor cells proliferative ability, *EAA* endothelial progenitor cells adhesion ability, *EMA* endothelial progenitor cells migration ability, *ETF* endothelial progenitor cells tubule forming ability, ∆ the change value of various index before and after treatment

### Correlation between change in endothelial progenitor cells, brachial–ankle pulse wave velocity, and other clinical parameters

**Fig. 5:**
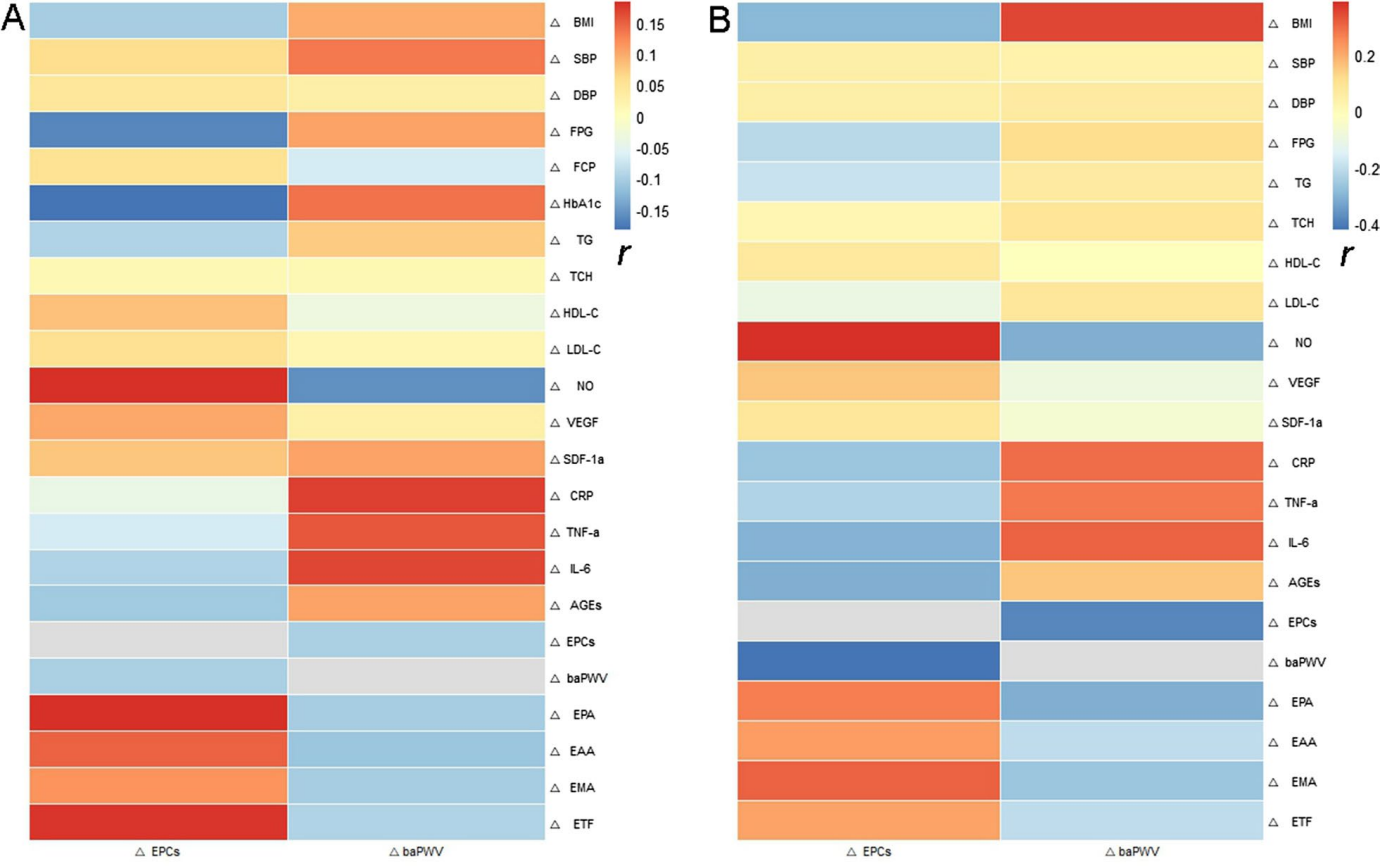
Pearson correlation analysis of the correlation between changes in EPCs and baPWV (ΔEPCs and baPWV) and changes in other clinical parameters (r). **a** MET group; **b** MET-DUL group. *BMI* body mass index, *SBP* systolic pressure, *DBP* diastolic pressure, *FPG* fasting plasma glucose, *FCP* fasting C peptide, *HbA1c*, glycated haemoglobin A1c, *TG* triglyceride, *TCH* total cholesterol, *HDL-C* high density lipoprotein cholesterol, *LDL-C* low density lipoprotein cholesterol, *NO* nitric oxide, *VEGF* vascular endothelial growth factor, *SDF-1α* stromal cell derived factor-1α, *CRP* C-reactive protein; *TNF-α* tumour necrosis factor-α, *IL-6* interleukin-6, *AGEs* advanced glycation end products, *EPCs* endothelial progenitor cells, *baPWV* brachial–ankle pulse wave velocity, *EPA* proliferative ability of endothelial progenitor cells, *EAA* adhesion ability of endothelial progenitor cells, *EMA* migration ability of endothelial progenitor cells, *ETF* tubule forming ability of endothelial progenitor cells; ∆ the change value of various index before and after treatment; r correlation coefficient

**Fig. 6:**
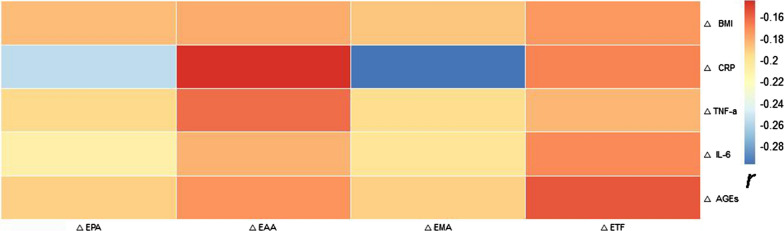
Pearson correlation analysis of ∆BMI, ∆CRP, ∆IL-6, ∆TNF-α, ∆AGEs with ∆ EPA, ∆EAA, ∆EMA, ∆ETF in the MET-DUL group (r). *MET-DUL* Metformin combined with dulaglutide treatment group; *BMI* body mass index; *CRP* C-reactive protein; *TNF-α* tumour necrosis factor-α; *IL-6* interleukin-6; *AGEs* advanced glycation end products; *EPA* proliferative ability of endothelial progenitor cells; *EAA* adhesion ability of endothelial progenitor cells; *EMA* migration ability of endothelial progenitor cells; *ETF* tubule forming ability of endothelial progenitor cells

Pearson correlation analyses showed that there was no correlation between ΔEPCs, ΔbaPWV, and the other observed index Δ-values of the MET group (*P* > 0.05; Fig. 5A). However, in the MET-DUL group, ΔEPCs were negatively correlated with ΔBMI (*r* = − 0.296, *P* = 0.013), ΔFPG (*r* = − 0.218, *P* = 0.042), ΔCRP (*r* = − 0.263, *P* = 0.019), ΔTNF-α (*r* = − 0.237, *P* = 0.028), ΔIL-6 (*r* = − 0.304, *P* = 0.011), ΔAGEs (*r* = − 0.311, 0.007), and ΔbaPWV (*r* = − 0.424, *P* < 0.001); and positively correlated with ΔNO (*r* = 0.389, *P* = 0.001), ΔEPA (*r* = 0.274, *P* = 0.017), ΔEAA (*r* = 0.219, *P* = 0.028), ΔEMA (*r* = 0.315, *P* = 0.009), and ΔETF (*r* = 0.211, *P* = 0.033; Fig. 5B). Moreover, ΔbaPWV positively correlated with ΔBMI (*r* = 0.353, *P* = 0.003), ΔCRP (*r* = 0.299, *P* = 0.008), ΔTNF-α (*r* = 0.283, *P* = 0.008), and ΔIL-6 (*r* = 0.315, *P* = 0.005); and negatively correlated with ΔEPCs (*r* = − 0.386, *P* < 0.001), ΔEPA (*r* = − 0.316, *P* = 0.009), ΔEAA (*r* = − 0.208, *P* = 0.036), ΔEMA (*r* = − 0.264, *P* = 0.012), and ΔETF (*r* = − 0.206, *P* = 0.041; Fig. 5B). In addition, ΔCRP, ΔTNF-α, ΔIL-6, and ΔAGEs were negatively correlated with ΔEPA and ΔEMA (*P* < 0.05; Fig. 6).

### Multiple linear stepwise regression analysis

In the MET-DUL group, ΔEPCs was the dependent variable and the other observed index Δ-values were the independent variables. ΔBMI, ΔNO, ΔCRP, and ΔIL-6 were determined as independently-related factors affecting ΔEPCs (*P* < 0.05; Table 4). Likewise in the MET-DUL group, ΔbaPWV was the dependent variable and the other observed index Δ-values were the independent variables. ΔNO, ΔIL-6, ΔEPCs, ΔEPA, and ΔEMA were determined as independently-related factors affecting ΔbaPWV (*P* < 0.05; Table 5).

**Table 4:** Multiple linear regression analysis of ΔEPCs with Δ value of other clinical parameters in the MET-DUL group

Variables	β	SE	t value	*P* value	95% CI
ΔBMI	− 0.217	0.097	− 3.328	0.019	− 0.351 to − 0.892
ΔNO	0.496	0.227	7.591	< 0.001	1.195 to 2.767
ΔCRP	− 0.198	0.116	− 2.793	0.042	− 0.184 to − 0.901
ΔIL-6	− 0.294	0.102	− 4.985	0.008	− 0.271 to − 0.849
ΔAGEs	− 0.227	0.175	− 3.849	0.025	− 0.327 to − 0.923

*MET-DUL* metformin combined with dulaglutide treatment group, *EPCs* endothelial progenitor cells, *BMI* body mass index, *NO* nitric oxide, *CRP* C-reactive protein, *IL-6* interleukin-6, *AGEs* advanced glycation end products, *∆* the change value of various index before and after treatment

**Table 5:** Multiple linear regression analysis of ΔbaPWV with Δ value of other clinical parameters in the MET-DUL group

Variables	β	SE	t value	*P* value	95% CI
ΔNO	− 0.257	0.115	− 3.287	0.026	− 0.286 to − 0.903
ΔIL-6	0.186	0.109	2.832	0.043	1.093 to 2.886
ΔEPCs	− 0.373	0.147	− 5.206	0.002	− 0.305 to − 0.849
ΔEPA	− 0.296	0.151	− 3.749	0.018	− 0.261 to − 0.919
ΔEMA	− 0.215	0.132	− 2.985	0.037	− 0.174 to − 0.863

*MET-DUL* metformin combined with dulaglutide treatment group, *baPWV* brachial–ankle pulse wave velocity, *NO* nitric oxide, *IL-6* interleukin-6, *EPCs*, endothelial progenitor cells, *EPA*, endothelial progenitor cells proliferative ability, *EMA*, endothelial progenitor cells migration ability, *∆* the change value of various index before and after treatment

## Discussion

Vascular elasticity is essential for maintaining the normal physiological functions of blood vessels; therefore, decreased arterial elasticity is a manifestation of vascular injury [25]. The number and function of EPCs are of great significance to maintain the integrity of the vascular endothelial structure and prevent ASCVD [26]. Some studies suggest that the decrease in arterial elasticity is closely related to the reduction in the number of circulating EPCs, impaired EPC function, chronic inflammatory state, and many cardiovascular risk factors such as diabetes [27, 28]. Under these conditions, endothelial cell proliferation and repair function are impaired, and the integrity of vascular endothelium is destroyed, resulting in increased arterial stiffness and an increased risk of thrombosis [29, 30]. At present, some antidiabetic drugs have been found to increase the number of EPCs in the peripheral blood of patients with diabetes, improve the function of EPCs, and have anti-inflammatory effects [14], but studies about their effects on vascular elasticity are limited.

In this study, we observed for the first time that the combination of metformin and dulaglutide significantly improved arterial elasticity; increased the number of EPCs and NO levels; decreased IL-6 levels; and improved EPCs’ proliferation, adhesion, migration, and tubule formation abilities in T2DM patients with poor blood glucose control with metformin alone. Multiple linear regression analysis showed that the change in the number of EPCs, EPCs’ proliferation and migration abilities, and NO and IL-6 levels were independent influencing factors of baPWV change. This phenomenon was not observed in T2DM patients treated with metformin alone. Therefore, considering our results and previous studies [27, 31], we concluded that dulaglutide can increase the number of EPCs in peripheral blood, reduce chronic inflammation, enhance the function of EPCs and endothelial cells, and then improve the elasticity of arterial vessels in patients with T2DM.

In recent years, an increasing number of studies have shown that GLP-1RAs can improve the cardiovascular outcome of patients with T2DM [32]. However, it remained unclear whether these benefits were related to GLP-1RAs’ effect on the number and function of EPCs. In the REWIND trial [5], nearly 69% of the participants did not have cardiovascular disease, and it was suggested that dulaglutide may have a primary preventive effect on ASCVD in patients with T2DM. But remarkably, in the present study, none of the participants had ASCVD. Interestingly, our results showed that the decrease in baPWV after treatment with dulaglutide in T2DM patients significantly correlated with the increase in the number of circulating EPCs and EPC proliferation and migration abilities. The multiple regression analysis revealed that the increase in the number of circulating EPCs and EPCs’ proliferation and migration abilities were independent predictors of the decrease in baPWV. Therefore, on the basis of this study and previous reports [33], we believe that increasing the number and function of circulating EPCs may be one mechanism in which dulaglutide repairs endogenous vascular injury and improves arterial elasticity. This hypothesis needs to be confirmed in further studies. In addition, we observed that in T2DM patients previously treated with metformin, the number and function of EPCs did not change when the dose of metformin was increased, which was consistent with our previous results [13].

Insufficiency and dysfunction of EPCs are related to cardiovascular risk factors such as hyperglycaemia, hyperlipidaemia, hypertension, and obesity [34, 35]. In this study, there were no significant differences in blood glucose, blood lipid, or blood pressure between the MET and MET-DUL groups at 0 and 12 weeks, which suggests that the increased number of EPCs in the MET-DUL group may be independent of the dulaglutide effects on blood glucose, blood pressure, and blood lipids. Available experimental evidence, together with a few pilot studies in humans, shows that GLP-1RAs are capable of ameliorating myocardial function and protect myocardiocytes from ischemic damage, independent of their glucose-lowering effects [36]. In addition, the BMI of the MET-DUL group participants decreased significantly after treatment with dulaglutide, as previously reported [37]. In contrast, MET group participants did not present a significant change in BMI after treatment. Multiple linear regression analysis showed that ΔBMI was an independent influencing factor of ΔEPCs, which indicates that the increase in the number of circulating EPCs caused by dulaglutide might be related to weight loss in patients with T2DM. Previous studies reported that weight loss by diet in overweight populations can improve the number of circulating EPCs [38]. Richards et al. [39] found that the number of EPCs in the peripheral blood of obese T2DM patients increased significantly after weight loss by gastric bypass surgery. Although the above studies are different from the present study in terms of subjects and intervention methods, they all confirm that weight loss is closely related to the increase in the number of EPCs in peripheral blood, therefore these results support our findings.

NO, VEGF, and SDF-1α are the main mediators that promote bone marrow EPCs mobilisation [40, 41]. In vitro studies found that glucagon-like peptide-1 improves proliferation and differentiation of EPCs via upregulating VEGF generation [42], and the upregulation of GLP-1 receptor could improve the dysfunction of EPCs in late hyperglycaemia by regulating SIRT1 expression [43]. In this study, dulaglutide had no significant effects on VEGF and SDF-1α levels in the peripheral blood of patients with T2DM, but it significantly increased NO levels. This change was an independent factor affecting the increase of EPCs in peripheral blood. In patients with newly-diagnosed T2DM, liraglutide can increase NO levels in peripheral blood [44]. Some scholars think that this may be related to GLP-1RAs promoting the activity of NO synthase and increasing NO levels in endothelial cells [45]. Therefore, we speculate that the increased number of EPCs due to dulaglutide may be closely related to the increased NO levels in the peripheral blood of patients with T2DM.

Previous studies discovered that treatment with exenatide, but not with liraglutide, was able to inhibit the apoptosis of EPCs and significantly increase the number of circulating EPCs, possibly through an antioxidative/anti-inflammatory effect [12, 46]. In vivo studies showed that the number of circulating EPCs (CD34^+^/KDR^+^) increased significantly in exendin-4-treated diabetic rats [47]. It was found that dulaglutide could inhibit the production of reactive oxygen species and protein carbonyls in human umbilical vein endothelial cells and inhibit the activation of NLRP3 inflammatory bodies under high glucose conditions [48]. Another study revealed that dulaglutide also attenuated lipopolysaccharide-induced cardiomyocyte injury by inhibiting inflammation and oxidative stress [49]. At present, CRP, IL-6, and TNF-α are recognized as representative markers of inflammatory factors in patients with diabetes [50], which can damage EPCs’ function [51], and reduce endothelial regeneration and vascular repair [52]. Persistent hyperglycaemia can promote the glycosylation of protein components and increase AGE production. When AGE binds to AGE receptors, it can induce oxidative stress and affect the activity and function of EPCs [53]. Our study found that dulaglutide could significantly reduce CRP, IL-6, TNF-α, and AGE levels in the peripheral blood of patients with T2DM. Pearson correlation analysis showed that the changes in CRP, IL-6, TNF-α, and AGEs before and after treatment significantly correlated with changes in the quantity, proliferation, and migration of EPCs. Multiple linear regression analysis showed that ΔCRP and ΔIL-6 were independent influencing factors of ΔEPCs, which suggests that the anti-inflammatory and antioxidant mechanisms of dulaglutide might also influence the increased number and function of EPCs in the peripheral blood of T2DM patients. Previous studies revealed that exendin-4 could ameliorate high glucose-induced EPC dysfunction through regulating the production of IL-6 via the SDF-1β/CXCR7-AMPK/p38-MAPK axis [54]. Another study found that exendin-4 could reduce endoplasmic reticulum stress and reactive oxygen species by inhibiting p38 MAPK pathway in the EPCs of diabetic mice under high glucose conditions, thereby improving cell survival and reducing apoptosis [55]. However, the mechanism of dulaglutide affecting EPCs’ quantity and function through anti-inflammatory and antioxidant effects needs to be further explored.

The main shortcomings of this study are as follows: (1) the study has a small sample size and a short observation time. At the same time, it is unable to confirm the causal relationship between the changes in the number and function of EPCs and their cardiovascular benefits. Therefore, the findings need to be confirmed by prospective cohort follow-up studies. (2) Most of the patients included in this study are overweight or obese T2DM patients without ASCVD. Therefore, it is necessary to further explore the effect of dulaglutide on peripheral blood EPCs and arterial elasticity in non-obese T2DM patients or T2DM patients with ASCVD. (3) Reactive hyperaemia index (RHI) and flow-mediated vasodilation (FDM) are valuable indexes to evaluate vascular endothelial function and predict the prognosis of cardiovascular disease [56]. Due to conditional limitations, we did not measure RHI and FDM while determining baPWV in subjects.

## Conclusions

This study found that dulaglutide can increase the number and function of peripheral blood EPCs in patients with T2DM and improve arterial elasticity. This effect may be related to weight loss, inflammation reduction, and elevated NO levels, suggesting that the regulation of EPCs and the improvement of cardiovascular risk factors may be a mechanism of cardiovascular protection of dulaglutide.

## Data Availability

All data generated or analysed during this study and supporting our findings are included and can be found in the manuscript. The raw data can be provided by corresponding author on reasonable request.

## Abbreviations

EPCs: Endothelial progenitor cells
T2DM: Type 2 diabetes mellitus
baPWV: Pulse wave velocity
CRP: C-reactive protein
IL-6: Interleukin-6
TNF-α: Tumour necrosis factor-α
AGEs: Advanced glycation end products
NO: Nitric oxide
ASCVD: Atherosclerotic cardiovascular disease
GLP-1RAs: Glucagon-like peptide-1 receptor agonists
HbA1c: Glycosylated hemoglobin A1c
REWIND: Researching Cardiovascular Events with a Weekly Incretin in Diabetes
VEGF: Vascular endothelial growth factor
SDF-1α: Stromal cell-derived factor-1 α
TG: Triglyceride
BMI: Body mass index
TCH: Total cholesterol
FPG: Fasting blood glucose
HDL-C: High-density lipoprotein cholesterol
LDL-C: Lipoprotein cholesterol
DAS-ELISA: Double-antibody sandwich enzyme-linked immunosorbent assay
MTT: 3-(4,5-Dimethylthiazol-2-yl)-2,5-dipheny-ltetrazolium bromide
DMEM: Dulbecco’s Modified Eagle Medium
EPA: Endothelial progenitor cells proliferative ability
EAA: Endothelial progenitor cells adhesion ability
EMA: Endothelial progenitor cells migration ability
ETF: Endothelial progenitor cells tubule forming ability
